# Generating Images with Physics-Based Rendering for an Industrial Object Detection Task: Realism versus Domain Randomization

**DOI:** 10.3390/s21237901

**Published:** 2021-11-26

**Authors:** Leon Eversberg, Jens Lambrecht

**Affiliations:** Chair Industry Grade Networks and Clouds, Technische Universität Berlin, Straße des 17. Juni 135, 10623 Berlin, Germany; lambrecht@tu-berlin.de

**Keywords:** data-centric AI, deep learning, domain randomization, image synthesis, object detection, physics-based rendering, synthetic images

## Abstract

Limited training data is one of the biggest challenges in the industrial application of deep learning. Generating synthetic training images is a promising solution in computer vision; however, minimizing the domain gap between synthetic and real-world images remains a problem. Therefore, based on a real-world application, we explored the generation of images with physics-based rendering for an industrial object detection task. Setting up the render engine’s environment requires a lot of choices and parameters. One fundamental question is whether to apply the concept of domain randomization or use domain knowledge to try and achieve photorealism. To answer this question, we compared different strategies for setting up lighting, background, object texture, additional foreground objects and bounding box computation in a data-centric approach. We compared the resulting average precision from generated images with different levels of realism and variability. In conclusion, we found that domain randomization is a viable strategy for the detection of industrial objects. However, domain knowledge can be used for object-related aspects to improve detection performance. Based on our results, we provide guidelines and an open-source tool for the generation of synthetic images for new industrial applications.

## 1. Introduction

Synthetic data are one of the most promising areas of research in modern deep learning as this approach tries to solve the problem of insufficient training data [1]. Compared to collecting and manually labeling real-world images, generating synthetic images is much faster and cheaper. Furthermore, synthetic images can reduce inherent dataset bias in real training sets [2] (e.g., underrepresented viewpoints [3]) by balancing data distribution. Compared to manually labeled datasets, which have been shown to contain numerous errors [4,5], synthetic datasets have no label errors and pixel-perfect label accuracy and consistency (e.g., for bounding boxes or segmentation masks). Lastly, for industrial applications, synthetic images can be generated based on already available 3D CAD models. For those reasons, synthetic images offer a solution to the problem of limited labeled data in industrial deep learning.

Synthetic data for deep learning have been shown to work well on different tasks and domains. Mixing real and synthetic data can improve deep learning model’s generalization, compared to using only one source of training data [1,6,7,8]. However, training with synthetic data for real-world applications suffers from a general machine learning problem called dataset shift, where training and test data come from different distributions [9]. More specifically, in computer vision the source domain consists of synthetic images and the target domain consists of real-world images. In order to overcome this so-called domain gap, different strategies for the generation of synthetic training images have been explored, such as compositing real images with a cut-and-paste approach [10], rendering images either with very high randomization [11] or in a photorealistic way [12]. Each of these strategies usually involves randomizing multiple parameters in different ways. However, there is still no consensus on the best way to generate training images for object detection tasks. Mayer et al. [13] examined the question of what constitutes good synthetic training data for optical flow and disparity estimation but their findings might not transfer to the high-level computer vision domain of object detection. In an industrial application, we want to detect a texture-less turbine blade at a manual working station in a shopfloor. With this exemplary use case we study the domain gap between synthetic images based on a 3D model and real-world images from a camera for an industrial object detection task.

In an industrial environment the surrounding is not completely random. Lighting, background contents and object colors are typically much less versatile compared to commonly used large-scale datasets, such as PASCAL VOC [14] or COCO [15]. This is especially true if the camera in the deployment environment is stationary. Additionally, industrial objects are often texture-less and can have highly reflective materials (e.g., metallic objects). Thus, we can use this domain knowledge to model the environment accordingly in a rendering software.

Physically based rendering (PBR) attempts to minimize the domain gap between synthetic and real images by rendering photorealistic images. Pharr et al. [16] define PBR as a rendering technique that attempts to simulate reality by modeling the interaction of light and matter according to the principles of physics. The best way to achieve this is by using a path tracing render engine. A path tracer sends out rays of light for each pixel in the image that bounce off surfaces until they hit a light source [6].

In this work, we used PBR to generate synthetic training images for a deep neural network to solve an industrial object detection task. We compared different levels of variability and realism for different aspects in the image generation pipeline.

1. We systematically generated multiple sets of PBR images with different levels of realism, used them to train an object detection model and evaluated the training images’ impact on average precision with real-world validation images.
2. Based on our results we provide guidelines for the generation of synthetic training images for industrial object detection tasks.
3. Our source code for generating training images with Blender is open source (https://github.com/ignc-research/blender-gen, accessed on 24 November 2021) and can be used for new industrial applications.

The paper is structured as follows: Section 2 outlines work related to the task of generating synthetic training images. Section 3 presents the methodology of this paper, in which the subsections give details on how to setup the rendering engine scene. In Section 4, we present our results where we compared different rendering approaches and evaluated the object detection performance on real-world test data. In Section 5, we discuss those results and their limitations. Finally, Section 6 summarizes our work and we give practical recommendations on how to create synthetic training images for new industrial object detection tasks.

## 2. Related Work

In the following section we briefly describe some of the different approaches that have been used to generate synthetic training images for deep learning models.

### 2.1. Cut-and-Paste

Georgakis et al. [17] generated training images by cropping real object instances with a segmentation mask and placing them on support surfaces in background images. Additionally, the scale of the object was adjusted to the depth of the selected position in the image and objects were blended in with the background. In a concurrent approach, Dwibedi et al. [10] also generated training images by cutting segmented object instances of household items, but then randomly pasting them onto background images. In contrast to Georgakis et al., they suggest that for object detection models local patch-level realism of the bounding box is more important than the global scene layout (i.e., objects do not have to be placed physically correctly on surfaces and the scale can be chosen randomly). A direct comparison of the two approaches on the GMU dataset [18] supports the theory of patch-level realism [10]. Dvornik et al. [19] used [10] as a baseline and compared it to their context model. With their context model, objects were only placed in specific locations in the background image where the visual context of the surroundings are realistic (e.g., planes are placed in the sky and trains on train tracks). Compared to random object placements the context guidance achieved considerably better mean accuracy results on the VOC2012 dataset.

### 2.2. Domain Randomization

Tobin et al. [20] explored the concept of domain randomization (DR) in order to bridge the gap between a low-fidelity simulation environment and the real world. Their idea behind DR is that the real world can be seen as just another random instance of a simulation environment. High variability in rendered images is achieved by using random camera and object positions, changing the lighting and using non-realistic textures. Tremblay et al. [11] used DR to train an object detection model for cars. They rendered different 3D models of cars with random textures on top of random background images. Furthermore, object poses, camera viewpoints, lighting and flying distractor objects were randomized. Additionally, standard data augmentation techniques, such as flipping and adding Gaussian noise, were utilized.

In [21] the idea of structured domain randomization (SDR) was introduced for a car detection task. SDR uses DR on objects but it keeps the structure of real images by generating realistic global scene layouts. For example, this means that cars are placed on roads and pedestrians on sidewalks. They argue that by using SDR the neural network can learn the relationship between cars and roads.

In [22] Hinterstoisser et al. used OpenGL to render 3D CAD models with Phong lighting [23] on top of real background images. The background images were taken with a camera in the highly cluttered test setting without the objects that are to be detected. Each object was positioned at a random location in the image with a randomly sampled scale and orientation. Furthermore, they used data augmentation techniques such as swapping the background image channels, different light colors, Gaussian noise and blurring with a Gaussian kernel. This approach is similar to the cut-and-paste approaches but it uses rendered objects instead of cutting them out of real images. In a follow-up publication [24] they used a slightly different method where every pixel in the background is composed of randomly sampled 3D models. Furthermore, they propose a deterministic schedule to sample the foreground objects poses in order to balance out the training image distribution. Lastly, they allow background objects to occlude up to 30% of the foreground objects.

### 2.3. Physics-Based Rendering

Compared to DR, which randomizes the full content of the simulated environment, PBR uses rendering randomization. PBR tries to simulate reality as close as possible while also randomizing environment parameters, such as lighting and the virtual camera’s position, to generate diverse training data [25]. Hodaň et al. [12] used the path tracing render engine Arnold [26] to generate highly photorealistic images in order to train a Faster-RCNN object detector. High realism was achieved by rendering 3D models of objects with realistic materials inside highly realistic 3D models of indoor scenes. The complex scenes were either purchased or created with the help of LIDAR scans, photogrammetry and artists using 3D modeling software. Furthermore, they used physics simulation to generate realistic object poses. When compared to the baseline of Hinterstoisser et al. [22], who rendered objects with OpenGL on top of images of the test scene, the realistic scenes achieved an improvement of up to 24% in mean average precision. With high quality render settings (average render time of 720 s per image) they got an improvement of 6% over low quality settings (15 s) on the LineMod-Occluded [27,28] dataset but no improvement on the Rutgers APC [29] dataset. Thus, they conclude that low-quality settings are sufficient for scenes with simple materials and lighting. Furthermore, they evaluated the importance of context. When objects were placed in a scene that realistically modeled the test data setup, they achieved an improvement of up to 16% compared to a scene that was out-of-context.

Rudorfer et al. [30] also experimented with placing objects inside a 3D modeled scene. The scene was created with Blender and consisted of a very simple setup with objects placed on top of a black box using physics simulation; however, the results showed that it was not possible to train a single shot pose [31] network with this scene setup. In contrast, they achieved much better results by rendering the objects without physics simulation on top of random background images. This suggests that a diverse background is much more important than physics-based object placement and that modeling a 3D scene environment is only beneficial when it is implemented in a highly photorealistic way. Additionally, they found that using background images of the test scene was superior to using random images from the COCO dataset.

In [3], V-Ray for 3ds Max was used by Movshovitz-Attias et al. to render 3D CAD Models of cars in order to train a viewpoint estimation network. To obtain diverse images they randomly sampled the light sources position, energy and color temperature. Furthermore, they used random background images from the PASCAL dataset [14] and a variety of parameters for the virtual camera. In their evaluation they show that a complex modeling of material and random directional lighting outperforms simple material and ambient lighting. They point out that the best cost-to-benefit ratio can be achieved with a high number of synthetic images combined with a small number of real images.

Jabbar et al. [32] used the software Blender to create photorealistic images of transparent drinking glasses. They used high dynamic range images (HDRIs) as 360 degree background images, which also provide complex image-based lighting (IBL) [33], thus removing the need to manually setup realistic lighting in the scene. Their results showed a substantial improvement when the background was similar to the test data, compared to using completely random backgrounds.

Wong et al. [34] published a synthetic end-to-end pipeline for industrial applications. First, texturized 3D object models were created with photogrammetry software. Then, synthetic images were created with Blender by randomly sampling camera position, point light source number and intensity and random background images from the SUN dataset [35]. Their pipeline shows that synthetic images can be used for deep learning even when there is no accurate 3D model available.

### 2.4. Domain Adaptation

In addition to the aforementioned approaches, domain adaptation techniques can be used to further bridge the domain gap between synthetic and real images. Generative adversarial networks (GANs) [36] can be used to transform generated synthetic images closer to the target domain [37,38,39,40]. Alternatively, both source and target domain can be transformed into an intermediate domain, e.g., with the Laplacian Filter [40] or the Pencil Filter [41,42].

### 2.5. Summary

The cut-and-paste approach requires real-world data with segmentation masks. Furthermore, lighting, object texture and object viewpoint are fixed by the cut-out data, which thereby limits the images that can be generated. The approach of DR is popular in the self-driving cars literature, where it is unfeasible to model every possible outdoor object. DR has the advantage that it can be highly automated as no domain knowledge has to be manually incorporated. If it is not possible to bridge the domain gap with synthetic data, domain adaption techniques can be used to post-process the generated images.

We presume that for industrial use cases, where the environment is known a priori and 3D CAD models are readily available, domain knowledge can be utilized to generate better training data than using full domain randomization. However, in some cases the level of photorealism is inversely correlated to the amount of image variability (e.g., background images and object textures). The related work section showed different approaches to generate synthetic training images, sometimes with contradicting results such as the theory of local patch-level realism [10] versus more realistic context models [19,21]. Table 1 compares selected PBR and DR approaches that were presented in this section and could be applied to an industrial object detection task. To summarize, there is no consensus on how to best generate synthetic images. Our work tackles this research gap by comparing a spectrum of different strategies that have been proposed in the literature. Our goal is to provide hands-on recommendations on how to generate synthetic training images for a specific object detection task based on a systematic evaluation of different approaches and parameters for PBR in an industrial use case.

## 3. Method

**Figure 1:**
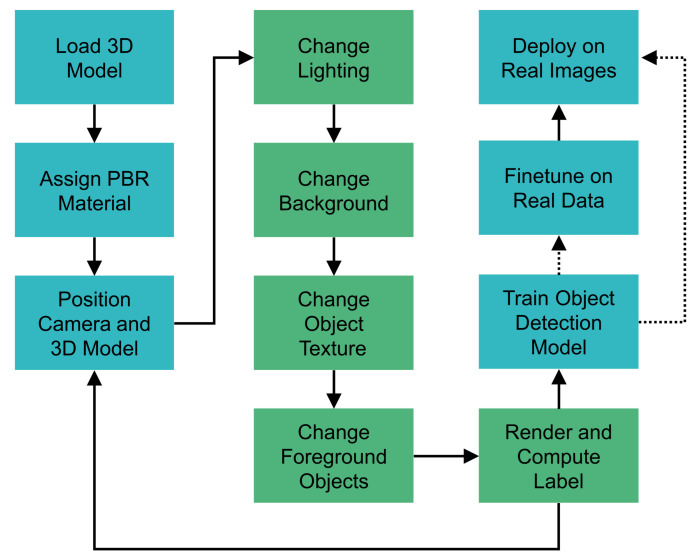
Our methodology for training an object detection model based on a 3D model as input. Different approaches for the green boxes are investigated in this paper.

We used the open-source 3D creation suite Blender to generate images and bounding box labels. Blender uses a path tracer rendering engine to generate physics-based renderings and can be fully automated using Python scripts. For those reasons, Blender is a popular tool amongst researchers for the automated generation of training images for deep learning (e.g., [30,32,34,44,45]). Our workflow and the scope of investigated aspects are depicted in Figure 1, given the constraint that the image generation pipeline can be easily adapted to new objects and industrial use cases. In this work, we compare different strategies on how to model lighting and the background in Blender. Furthermore, we investigate if different object textures or adding occluding foreground objects can improve the detection model’s performance. Following the theory of patch-level realism [10], we do not model physically correct object placement in the global scene layout. Details on how we set up the Blender scene for image generation as well as the object detection model are described in the following subsections.

### 3.1. 3D Object Model

**Figure 2:**
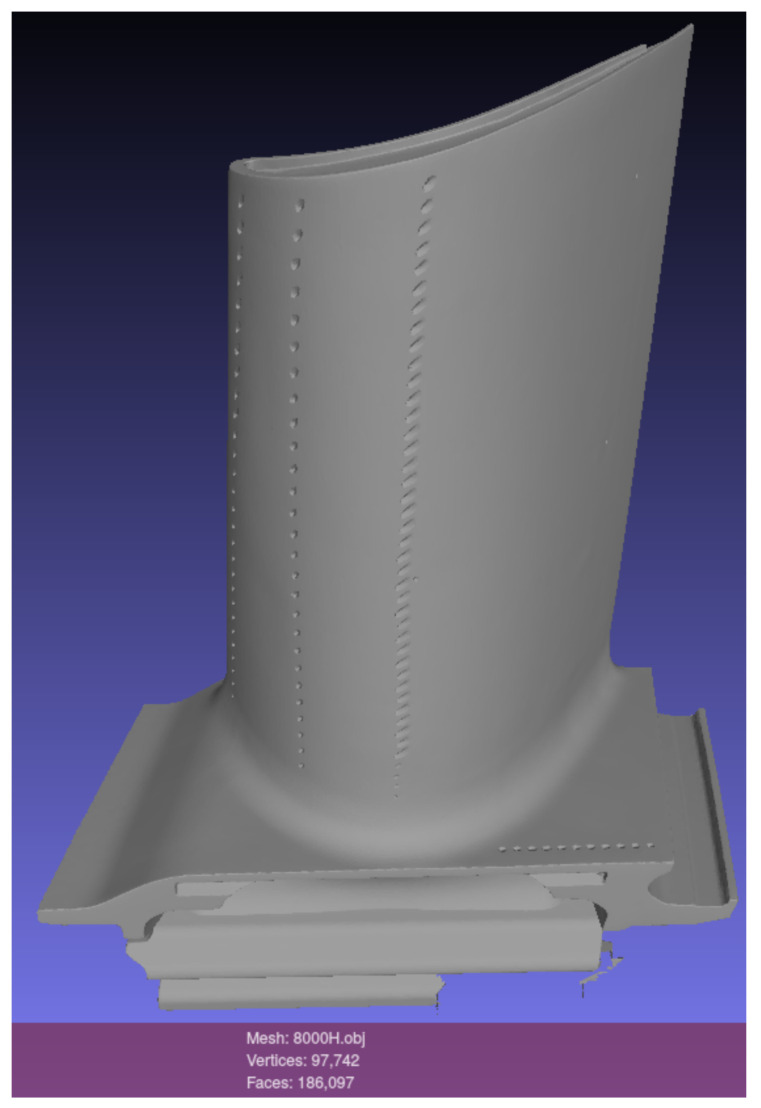
3D model of a turbine blade, obtained from an industrial 3D scanner.

Industrial objects are often texture-less and their visual appearance is thus defined by their shape, color, reflectance and the environment’s lighting [46]. Transferring methods evaluated on existing public datasets to specific industrial scenarios can lead to quite different results [47]. Therefore, we used a texture-less turbine blade and created our own training and evaluation images at a manual working station in a shopfloor environment. We obtained a 3D model of the turbine blade from an industrial 3D scanner (see Figure 2) and imported the file to Blender where it was placed in the scene’s origin. We assigned the model a physics-based material according to the bidirectional scattering distribution function (BSDF), which describes the scattering of light on surfaces [16]. We changed values for the material properties Color, Roughness, Specular and Metallic in Blender to simulate real-world appearance.

### 3.2. Positioning of Camera and 3D Object

The camera position in spherical coordinates, denoted by the radius $r\in R^{+}$, inclination θ∈[0,π] and azimuth ϕ∈[0,2π], is transformed with (1) into Cartesian coordinates $\left(x_{c},y_{c},z_{c}\right)$ and then placed in the scene.

(1) (x,y,z)=(rcosϕsinθ,rsinϕsinθ,rcosθ)

**Figure 3:**
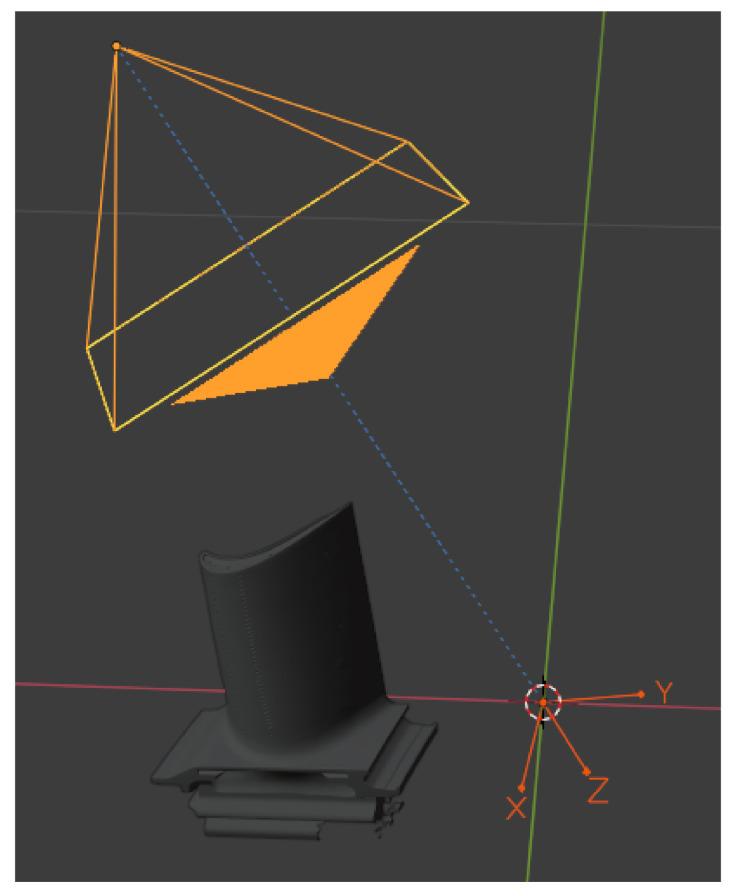
The camera is constrained to look at an invisible object (X, Y, Z) at the scenes origin. By moving the 3D model and rotating the camera through the empty object we change the pose of our 3D model in the rendered image.

By sampling uniformly between minimum and maximum values, the camera is randomly positioned on a spherical shell around the origin of the Blender scene. The radius *r* controls the scale of the object in the rendered image. By limiting ϕ and θ certain viewpoints can be excluded (e.g., the view of the object from below). By uniformly sampling the object’s position our 3D model of the turbine blade moves away from the image center. Furthermore, we constrain the camera to always look at an invisible object which is positioned at the scene’s origin. We sample three rotation angles $\alpha_{1,2,3}∼U\left(0,2\pi \right)$ and perform a XYZ Euler rotation on the invisible object in order to randomly rotate the constrained camera and thus create more diverse training data. The placement of the constrained camera, 3D model and the invisible object is depicted in Figure 3. If more than 10% of the 3D model’s bounding box is outside of the rendered image, the scene setup is automatically resampled.

### 3.3. Modeling of Lighting

We compared two different approaches of modeling lighting: point lights and image-based lighting.

#### 3.3.1. Point Lights

**Figure 4:**
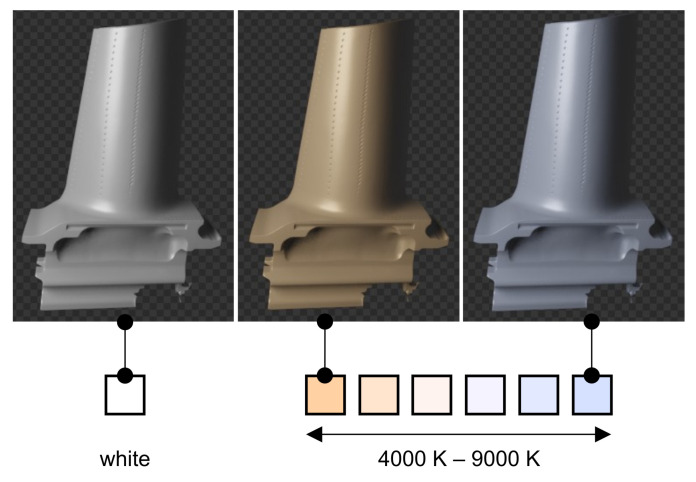
Each point light color is randomly sampled from six discrete values with color temperatures ranging from warm 4000 K to cool 9000 K in addition to white light. Ref. [49] was used for color conversions.

Point lights create omnidirectional light originating from one point. This creates illuminated areas on the 3D model. We create a randomly sampled number of point lights $n_{PL}∼U\left\{n_{min},n_{max}\right\}$. Each point light’s location is sampled according to (1) with the same parameters as the ones for the camera. Each light has a power of $E_{PL}∼U\left(E_{min},E_{max}\right)$. In a simple baseline we only use lights with white color. Additionally, in a more complex approach we randomly sample each light’s color temperature from a realistic range consisting of six discrete values in addition to white light. As depicted in Figure 4, the color temperatures range from warm 4000 K to cool 9000 K (natural daylight is around 5000 K [48]). Modeling realistic lighting with point lights is not an easy task because the number of lights, their distance to the 3D model and their power are all interrelated hyperparameters.

#### 3.3.2. Image-Based Lighting with HDRIs

**Figure 5:**
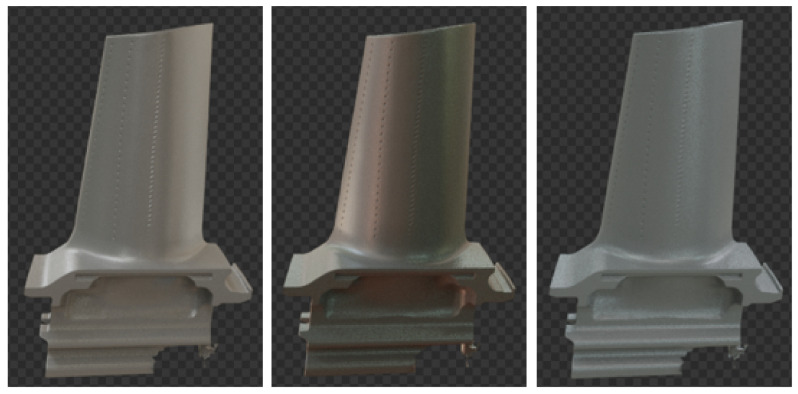
Compared to point lights, image-based lighting with an HDRI creates a more balanced ambient illumination. The images were rendered with three different HDRIs with $E_{IBL}=1$.

HDRIs provide 360 degrees of image-based lighting, thus there is no need to manually model any additional light sources. Compared to point lights or other directed light sources, the easy setup of IBL is a major advantage. Furthermore, IBL also enables objects to have realistic reflections and translucency. We used 123 different indoor HDRIs in 4K resolution (we used all available indoor HDRIs from https://polyhaven.com/hdris, accessed on 6 September 2021) as background environment textures. Three examples are shown in Figure 5. We uniformly sample the HDRI light emission strength $E_{IBL}∼U\left(E_{min},E_{max}\right)$ to create diverse training data.

### 3.4. Modeling of the Background

We compared three different approaches of generating the image background: random images from a large-scale dataset, 360 degree environment textures and taking pictures of the application domain.

#### 3.4.1. Random Background

For each generated scene we randomly selected a background image from the COCO 2017 train dataset [15], which consists of more than 118,000 images. We rendered only the 3D objects in the scene and then composited this with the random background image. The background image was cropped to fit the rendered image size.

#### 3.4.2. HDRIs

Similar to the random COCO background, in this approach we use the 123 indoor HDRIs in 4K resolution, which were also used for image-based lighting, as background images. HDRI environment maps provide a dynamic 360 degree background (i.e., the background is based on the scene’s virtual camera angle). Furthermore, our selected indoor HDRIs have a more realistic indoor environment compared to the full COCO dataset, which also includes outdoor images.

#### 3.4.3. Images of the Application Domain

**Figure 6:**
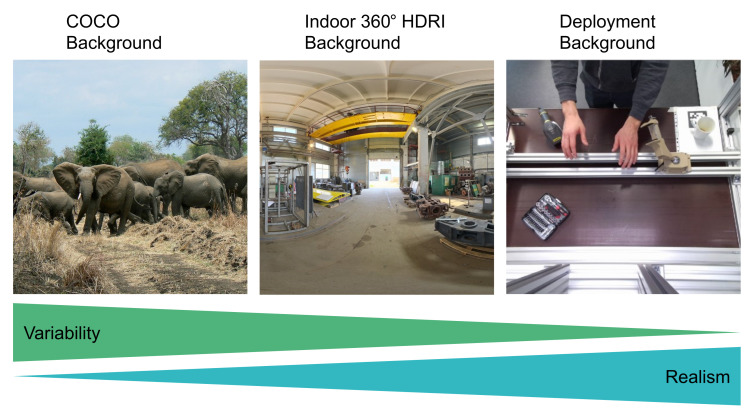
The three different types of background images which were used. With these three choices we investigate the trade-off between image variability and level of realism.

Lastly, we took pictures from the application domain where our model will be deployed. We collected 43 images with different levels of illumination of the working area in which we want to detect the real turbine blade. In each image we changed the position of some elements, such as a mug or a box with tools. While these images provide a very realistic background compared to COCO images, they also strongly limit the variability of the background. As shown in Figure 6, the different approaches of modeling the background have different image variability (based on number of images and image content diversity) and realism (based on domain knowledge of the application domain).

### 3.5. Object Texture

**Figure 7:**
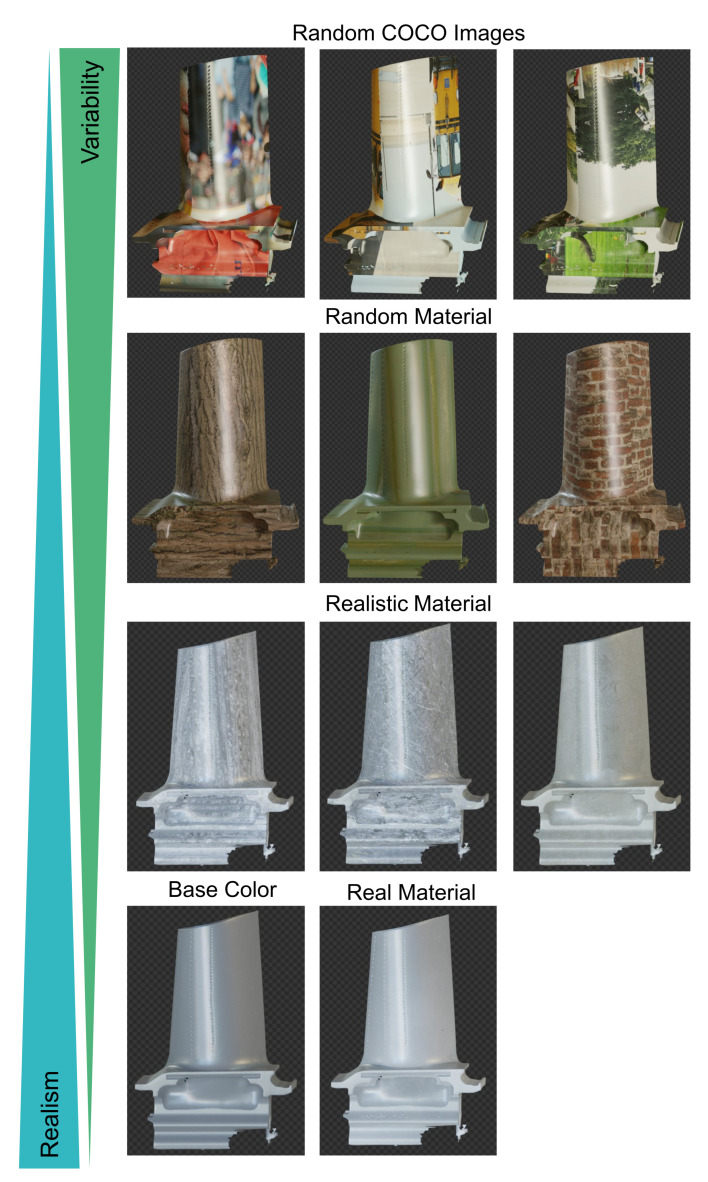
Examples of different textures that were used. We compare random COCO images, random materials, realistic materials, real material created from photographs of the turbine blade and a single base color against each other.

Randomizing object textures is a feature that is heavily used in DR. As shown in Figure 7, we compare our simple model with a grey base color to random textures, realistic textures and real textures that we created ourselves. While the real textures have the highest amount of realism, random textures provide more variability in the training data. For random textures, we used the COCO 2017 train images as well as 220 different material textures from https://polyhaven.com/textures, accessed on 6 September 2021. For realistic textures, we manually selected 55 different textures from https://www.textures.com and https://polyhaven.com/textures, accessed on 6 September 2021, that provide a realistic, yet slightly different texture compared to the real object’s texture. These include textures from greyish concrete, bare metal, plaster and rock. We also took 20 close-up pictures in slightly different lighting conditions with a smartphone camera from the turbine blade’s surface and created our own textures out of them. For every rendered image, we sampled one image texture from the chosen pool of textures.

### 3.6. Adding Foreground Objects

**Figure 8:**
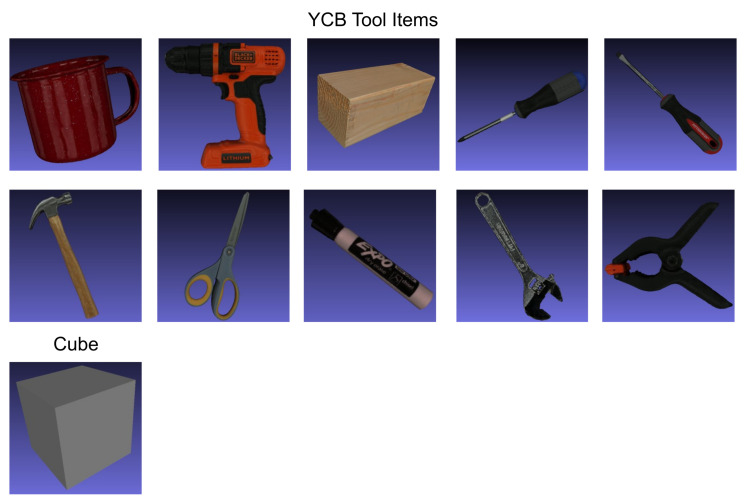
Ten realistic YCB tool items [50] are used as additional foreground objects and compared to a simple cube.

We added a pool of distracting 3D object models, depicted in Figure 8, consisting of the YCB dataset’s [50] tool items (licensed under CC BY 4.0): mug, power drill, wood block, Phillips screwdriver, flat screwdriver, hammer, scissors, large marker, adjustable wrench and medium clamp (We used the 64k laser scans from http://ycb-benchmarks.s3-website-us-east-1.amazonaws.com, accessed on 6 September 2021). We used those tools because they fit within our industrial context. Furthermore, we compared the YCB tools to simple cubes as foreground objects. While cubes are less realistic, they offer perfectly flat surfaces, which enables better texture mapping. For every rendered image we sample $n_{FG}∼U\left\{n_{min},n_{max}\right\}$ distracting foreground objects. Then, we randomly moved and rotated those objects within the scene. These distracting objects add the concept of occlusion when they are randomly sampled in front of the turbine blade. Furthermore, by adding additional 3D objects the object detection model cannot rely on artifacts that are introduced by the rendering engine compared to the composited background image.

In addition to the YCB dataset textures, we also randomized the distracting objects’ textures. Following the methodology of Section 3.5, we explored random COCO images and random material textures.

### 3.7. Computation of Bounding Box Labels

After setting up the scene in Blender, we transformed all vertices of the turbine blade’s 3D model from world space $\left(x_{i},y_{i},z_{i}\right)$ to rendered image space $\left(u_{i},v_{i}\right)$ according to (2), which uses the virtual camera’s projection matrix $P\in R^{3\times 4}$ and a scaling factor *s*.

(2) $$s\cdot \left(\begin{array}{c} u_{i}\\ v_{i}\\ 1 \end{array}\right)=P\cdot \left(\begin{array}{c} x_{i}\\ y_{i}\\ z_{i}\\ 1 \end{array}\right)$$

After transforming all vertices, we use the obtained minimum and maximum values $\left\{u_{min},u_{max},v_{min},v_{max}\right\}$ to generate tight bounding boxes (x,y,w,h) around the rendered turbine blade according to (3) and (4) in the COCO data format.

(3) $$\left(x,y\right)=\left(u_{min},v_{min}\right)$$

(4) $$\left(w,h\right)=\left(u_{max}-u_{min},v_{max}-v_{min}\right)$$

**Figure 9:**
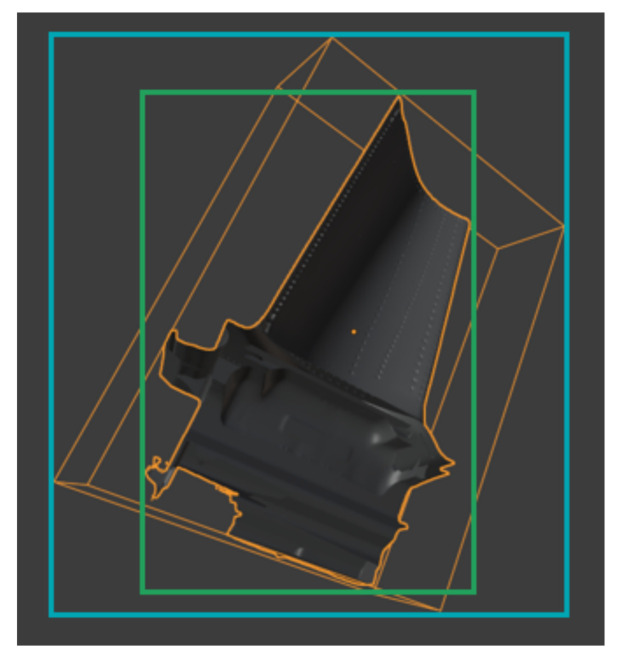
Comparison of bounding box computations. Using the eight 3D bounding box coordinates results in the blue label and using all mesh vertices results in the green label.

Figure 9 shows the difference in the resulting 2D bounding box label when using (2)–(4) on all vertices of the 3D model versus using only the 3D bounding box coordinates provided by Blender. Though using all vertices is more computationally expensive, the resulting labels are much tighter than the ones from the 3D bounding box.

### 3.8. Object Detection Model and Training

**Figure 10:**
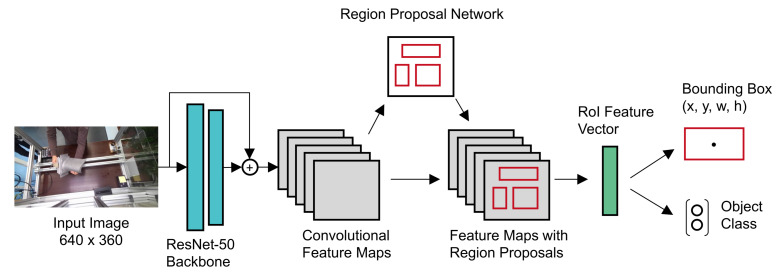
Object detection model based on Faster R-CNN.

For our object detection task we used a Faster R-CNN network [51], as shown in Figure 10. We used a pre-trained ResNet-50 backbone [52] to obtain convolutional feature maps. These feature maps are used by a region proposal network (RPN), which outputs regions of interest (RoI) as rectangular bounding boxes and classifies them as either being an object or not. Given the convolutional feature maps from the CNN backbone and the RoIs from the RPN, a feature vector is computed for each RoI. Then, the model outputs discrete probabilities for each object class as well as bounding boxes [53]. Each bounding box prediction (x,y,w,h) is defined by its upper-left corner position (x,y) in the image, its width *w* and its height *h*. While bigger or newer models might increase the final detection result, we chose to keep the model fixed following a data-centric AI approach and only change the generated input images.

As evaluation metric for our object detection task we use average precision (AP), which approximates the area under the precision/recall curve. Precision and recall are defined by true positives tp, false positives fp and false negatives fn according to (5) and (6). A detection is considered a true positive if the Intersection over Union (IoU) of a predicted bounding box with the ground truth bounding box exceeds a given threshold [54]. With the PASCAL VOC metric this threshold is 50% ($AP^{0.5}$). For the COCO metric an average over 10 IoU thresholds is computed, ranging from 50% to 95% with a step size of 5% ($AP^{\left[0.5:0.95\right]}$).

We trained every model for a maximum of 25 epochs. Then, the model with the highest $AP^{\left[0.5:0.95\right]}$ was selected. More details on our choices for deep learning hyperparameters can be found in Table 2.

(5) $$precision=\frac{tp}{tp+fp}$$

(6) $$recall=\frac{tp}{tp+fn}$$

### 3.9. Validation Data

We recorded 650 validation images with a Microsoft Azure Kinect camera in 1080P resolution from the manual working station. In every image the turbine blade is visible and the pose of the turbine blade is different for all images. We allowed small forms of occlusion from a hand, fingers or from a vise. The validation images are as close as possible to the real industrial working conditions. We manually labeled the bounding box of the turbine blade for all images, which took about 6 s per image. By comparing validation error on real images for different image generation strategies, we can measure which strategy is best suited to close the domain gap between synthetic images and real-world images.

## 4. Experiments and Results

If not otherwise specified, the following results were created by rendering 5000 images and then training the object detection model for 25 epochs (always using the same random seed for image generation and model training). We used point lights for lighting, random COCO background images, a grey base color for the turbine blade’s model and no additional foreground objects as an initial baseline. Starting with this baseline, we compare the different image generation approaches described in Section 3.3, Section 3.4, Section 3.5, Section 3.6 and Section 3.7.

On our computer with two Tesla M60 GPUs and an Intel Broadwell CPU, rendering took between 1.7 s and 5.6 s per image on average, depending on the scene configuration. Training Faster R-CNN with 5000 synthetic training images for 25 epochs took around 8 h on a single GPU.

### 4.1. Computation of the Bounding Box

**Figure 11:**
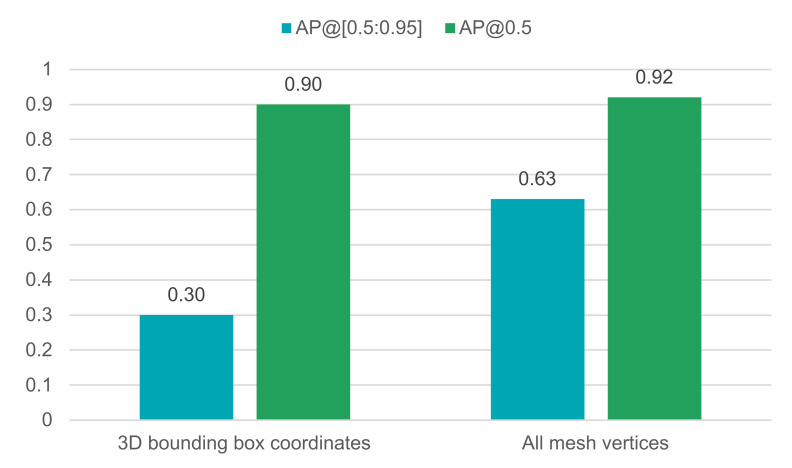
Comparison of bounding box label computation.

First, we compare two different strategies for computing the bounding box. As shown in Figure 11, computing tight bounding boxes by transforming all mesh vertices to image space leads to a much better performance in $AP^{\left[0.5:0.95\right]}$ than using 3D bounding box corner coordinates. When transforming all mesh vertices with Equations (2)–(4), rendering an image and computing the label took 3.3 s. On contrast, when transforming only the 3D bounding box coordinates to image space the process took only 1.7 s on average. However, bounding box computation time can be reduced by downsampling the number of vertices of the 3D mesh.

### 4.2. Lighting

The results for different lighting models are shown in Table 3. Adding color to point lights by randomizing color temperature improved the performance slightly compared to white point lights. Image-based lighting with 123 indoor HDRIs achieved the best performance while at the same time requiring less parameter choices.

### 4.3. Background

Table 4 shows that 360 degree indoor HDRIs are not a good choice for background images. We believe this is due to the position and orientation of the virtual camera, which is often looking towards the indoor ceiling or floor of the scene. For this reason, HDRIs often do not provide rich background images.

Furthermore, the high variability in the large-scale COCO dataset outperformed the high realism of domain-specific background images of the manual working station. Contrary to our initial belief, mixing COCO and deployment background images did not result in an improvement over using only COCO images.

### 4.4. Object Texture

Results for changing the turbine blade’s texture are shown in Table 5. Selecting textures with a realistic color palette achieved the best performance. Realistic material textures provide realism as well as more variability than the real material texture.

Random material textures performed only slightly worse; therefore, domain randomization of the object texture seems to be a viable alternative if no appropriate material textures are available. Projecting random COCO images onto the turbine blade’s UV map resulted in unnatural and irregular textures and thus performed the worst.

### 4.5. Foreground Objects

Table 6 shows the results of adding additional foreground objects. For the YCB tools, rendering up to three objects improves the detection performance slightly. Randomizing the YCB objects’ textures performed worse than using the original textures. We believe this is due to the fact that the YCB tools already have complex textures, thus there is no benefit in randomizing them.

The cubes offer perfectly flat surface areas, which are ideal for mapping textures onto them. As a result, using cubes as simple geometric shapes with random textures resulted in a slightly better AP than the YCB tools and a significant increase compared to no foreground objects.

### 4.6. Number of Rendered Images

**Figure 12:**
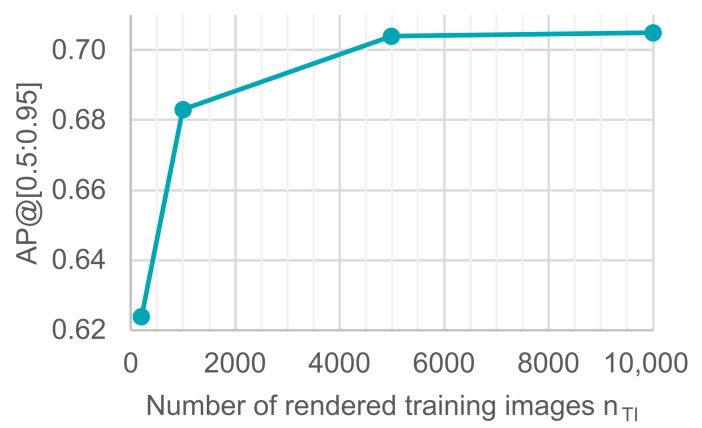
The impact of adding more synthetic training images on the object detection model for a training time of up to 24 h.

After investigating the image generation methodology in Blender, we investigated the number of rendered training images. Previous results used only 5000 rendered images and 25 epochs for training to reduce computation time while searching for optimal hyperparameters. Other than using more or less training data, we also changed the number of training epochs. All of the following models were trained for up to 24 h. Figure 12 shows the relationship between the number of rendered images for the training set and the average precision of the object detection model on validation images measured in $AP^{\left[0.5:0.95\right]}$. While the generation of synthetic data has the capability to create an unlimited amount of training data, the chart shows that a maximum average precision of $AP^{\left[0.5:0.95\right]}=0.7$ is reached already with $n_{TI}=5000$ training images. Adding more training data does not improve model performance after this point.

**Figure A1:**
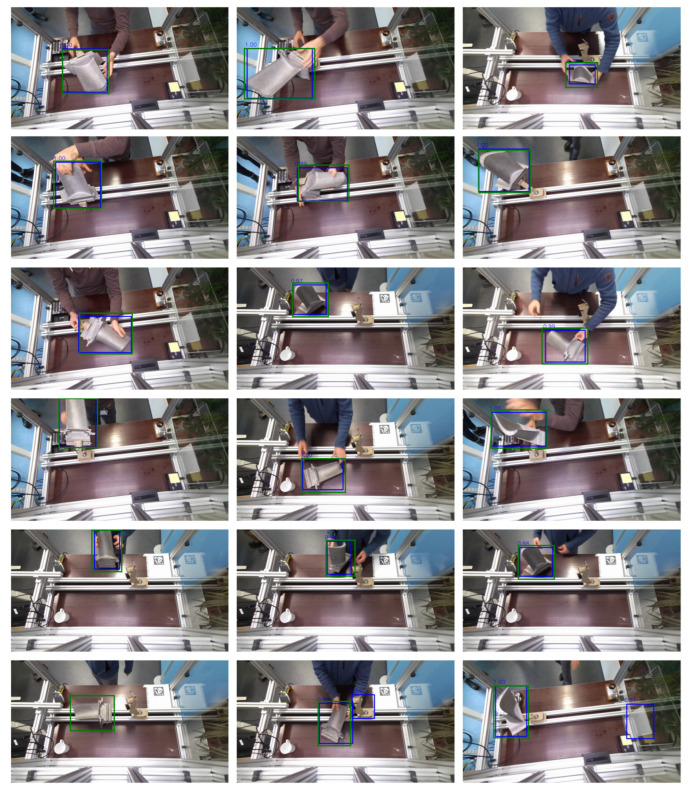
Object detection results on real validation data from our Faster R-CNN model trained purely on 5000 synthetic PBR images. Predictions are blue (including detection confidence) and manually created ground truth labels are green. The last row shows some errors.

Qualitative object detection results on validation data are depicted in Figure A1.

### 4.7. Using Real Images

In order to compare our PBR-based approach to real images, we also trained the Faster-RCNN object detection model with a small number of real images. Thus, we captured and labeled $n_{TI}=200$ images from the application domain in the same way as the validation data. Because it has been shown that training on synthetic data and then fine-tuning with real data in a two-step approach can achieve better performance than simply mixing synthetic and training datasets [7,8], we also used our PBR-model trained on $n_{TI}=5000$ images as a pre-trained baseline and fine-tuned this with the same 200 real images.

As shown in Table 7, the models trained only on synthetic PBR images or real images achieve the same performance. Furthermore, the fine-tuned model has a substantially higher average precision than the other two models. The model pre-trained on PBR images acts as a strong base for further fine-tuning on real images.

### 4.8. Transfer to New Objects

**Figure 13:**
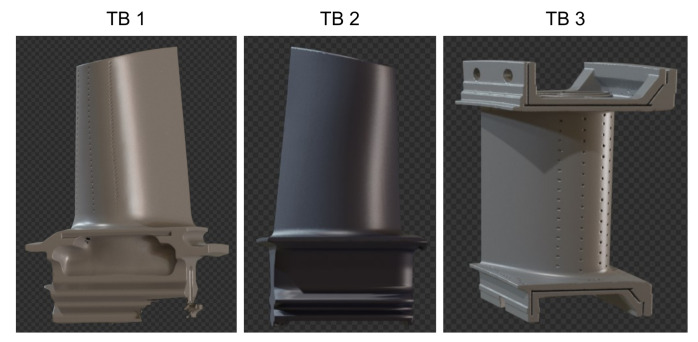
In addition to the previously studied TB 1, TB 2 and TB 3 are added as new test objects.

Finally, after thorough investigation of the image generation methodology and hyperparameters, we created three new test datasets with 200 test images each according to Section 3.9 with occlusion and clutter. In addition to our previous turbine blade (TB 1) we added two new objects (TB 2 and TB 3). The two new turbine blades differ significantly in color and geometry from our previously used model, see Figure 13. For all three objects we performed a sequential ablation study on the new test data. Table 8 shows that our image generation methodology can be transferred to new objects. Applying previous results on new objects resulted in an increase in $AP^{\left[0.5:0.95\right]}$.

### 4.9. Qualitative Results

**Figure A2:**
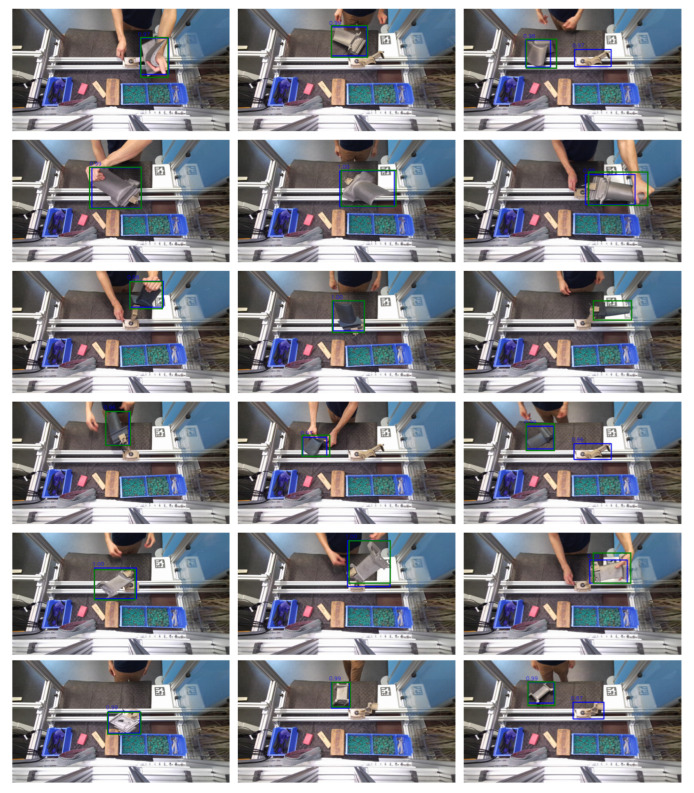
Object detection results on real test data from our three Faster R-CNN models trained purely on 5000 synthetic PBR images each. Predictions are blue (including detection confidence) and manually created ground truth labels are green. The last column shows some errors.

**Figure A3:**
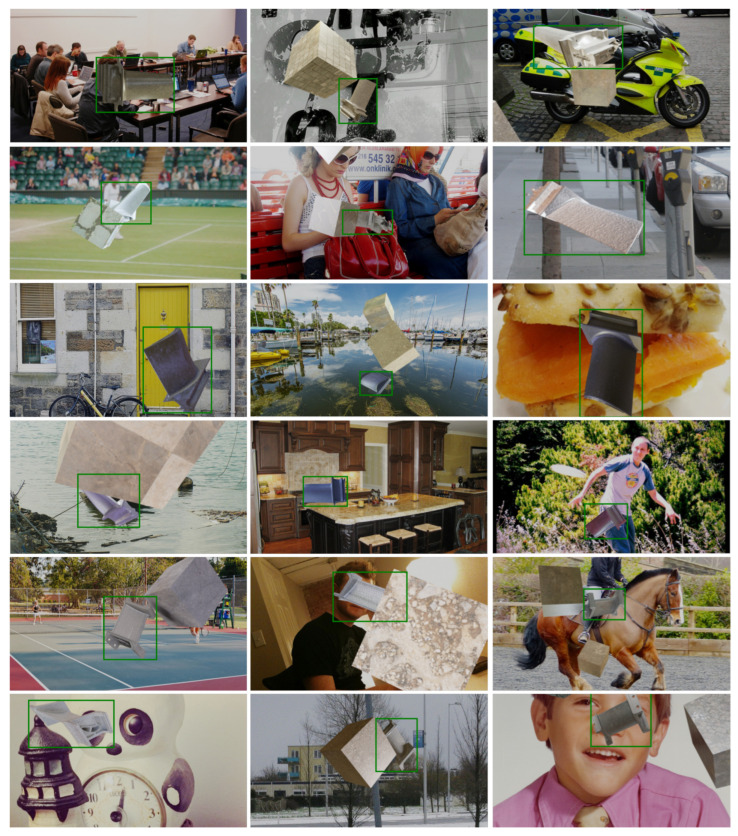
Examples of our synthetic training images generated with PBR. For the three turbine blades we used COCO background images, image-based lighting from HDRIs, random realistic material textures and up to three cubes with random material textures as additional foreground objects.

Qualitative results of our final object detection models for TB 1, TB 2 and TB 3, trained only on PBR-images, are shown in Figure A2. Our deep learning model usually detects the turbine blade with very high confidence and outputs a tight bounding box. Rarely observed errors are mostly images with high occlusion and false positive detections. Examples of the rendered training images are shown in Figure A3.

## 5. Discussion

The results from Section 4.2, Section 4.3, Section 4.4 and Section 4.5 can be arranged into two groups: object-related and non-object-related aspects. Background images and additional distracting foreground objects are unrelated to the object of interest. For both of these aspects the concept of domain randomization outperformed higher realism. Our results show that there is no need to use realistic image backgrounds or realistic distractor objects. On the other hand, lighting and object textures affect the visual appearance of the 3D model. For these aspects we found that realistic indoor lighting and realistic material textures performed the best. Although, random material textures resulted in the same $AP^{\left[0.5:0.95\right]}$ and a higher $AP^{0.5}$ than real material textures. This suggests that high variability is still an important aspect when trying to achieve high photorealism that should not be neglected.

However, our results on object texture and lighting are limited by the appearance of the turbine blade (TB 1) and the manual working station. As can be seen in the validation images from Figure A1, the turbine blade has a mostly homogenous grey color that is similar to other elements in the validation images and there was mostly artificial white light from above the table.

Even though we transferred our method to new objects (TB 2 and TB 3) and new test data in Section 4.8, our results are still limited by our specific use case. However, we provide the methodology and open-source tool to easily generate labeled PBR images for new objects and different industrial environments.

## 6. Conclusions

In this work we presented an image generation pipeline based on PBR that can generate synthetic training images for deep learning object detection models. With purely synthetic images as input data and thereby no manual labeling, we trained a Faster R-CNN model for texture-less turbine blades. We showed that the biggest improvements in average precision come from a tight bounding box label computation and optional fine-tuning on a small amount of real-world data. Furthermore, we evaluated different approaches regarding lighting, background images, object texture and additional foreground objects. Additionally, we transferred our methodology to new test data with two additional turbine blades and confirmed the positive effects of our image generation pipeline. Based on our results we propose the following guidelines for the generation of PBR training images for industrial objects.

First, we recommend image-based lighting by using HDRIs as environment textures. In addition to a slightly better average precision than point lights, IBL is much easier to setup with the only hyperparameter being the light’s emission strength. Second, we recommend using background images from a large-scale dataset, such as COCO. We showed that random background images perform better than a small amount of realistic images from the application domain. Third, we recommend randomizing the 3D object’s texture while at the same time keeping object appearance realistic. Lastly, we recommend using simple cubes with random material textures as additional distracting foreground objects. Based on our results, there is no need to use application-specific 3D foreground objects. Finally, we recommend rendering at least 5000 images per class as a starting point.

Our best image generation pipeline requires only the manual selection of realistic object textures. Background images, lighting and foreground objects are randomized from a pool of files that only need to be downloaded once. However, random object textures performed not much worse than realistic object textures and are therefore a viable alternative if realistic object textures are unavailable. With full domain randomization, industrial object detection models can be trained automatically based only on a 3D model and without the need of any domain knowledge.

For future research, we encourage others to try our open-source image generation pipeline in Blender (https://github.com/ignc-research/blender-gen, accessed on 22 November 2021) for new objects in different industrial environments as well as add further extensions to the image generation methodology. Additionally, alternative object detection models (e.g., YOLO or transformer-based models) could be used and compared to Faster R-CNN. Furthermore, domain adaptation techniques could be applied to further decrease the domain gap. While our work focused on the task of object detection, we believe that the methodology can be transferred to similar high-level computer vision tasks, such as object pose estimation or object segmentation.

## Figures and Tables

**Table 1:** Comparison of state-of-the-art image generation with PBR and DR.

	Lighting	Background	Object Texture	Occlusion	Object Placement
Hodaň et al. [12] 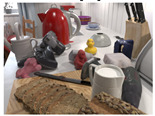	Standard light sources (e.g., point lights) and Arnold Physical Sky [26]	3D scene models	From 3D model	Multiple 3D objects	Physics simulation
Rudorfer et al. [30] 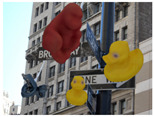	White point lights	Application domain images or COCO	From 3D model	Multiple 3D objects	Random
Movshovitz-Attias et al. [3] 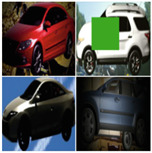	Directed light w/ different light temperatures	PASCAL	From 3D model	Rectangular patches	Random
Jabbar et al. [32] 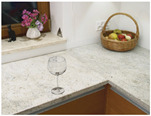	IBL from HDRIs	360° HDRIs	Glass material	None	On a flat surface
Wong et al. [34] 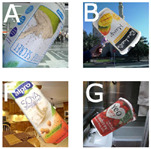	White point lights	SUN	From 3D model	None	Random
Tremblay et al. [11] 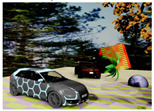	White point lights and planar light	Flickr 8K [43]	Random (Flickr 8K)	Multiple 3D geometric shapes	On a ground plane
Hinterstoisser et al. [22,24] 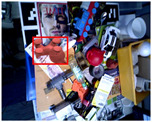	Random phong light with random light color	Application domain images or random 3D models	From 3D model	None or multiple 3D objects	Random

**Table 2:** Deep learning hyperparameters.

Hyperparameter	Value
Optimizer [55]	Stochastic gradient descent (SGD) with learning rate ϵ=0.00001, momentum μ=0.9, L^2^ weight decay α=0.0001
Epochs	25
Training examples	5000
Batch size	8
Image size	640 pixel × 360 pixel

**Table 3:** Lighting results.

Lighting Model	$n_{\mathrm{PL}}$	*E*	${\mathrm{AP}}^{\left[0.5:0.95\right]}$	${\mathrm{AP}}^{0.5}$
PL (white)	U{1,4}	U(20W,100W)	0.623	0.917
PL (white)	U{1,5}	U(20W,100W)	0.633	0.908
PL (white)	U{1,6}	U(20W,100W)	0.632	0.919
PL (white)	U{1,6}	U(20W,70W)	0.633	0.914
PL (white)	U{1,6}	U(20W,130W)	0.631	0.928
PL (temperature)	U{1,6}	U(20W,130W)	0.635	0.929
IBL with HDRIs	-	U(0.5,6)	0.640	0.926
IBL with HDRIs	-	U(0.5,7)	0.642	0.926
IBL with HDRIs	-	U(0.5,8)	0.641	0.931
IBL with HDRIs	-	U(1,8)	**0.642**	**0.931**

**Table 4:** Background results.

Background Model	${\mathrm{AP}}^{\left[0.5:0.95\right]}$	${\mathrm{AP}}^{0.5}$
COCO images	**0.642**	**0.931**
HDRI images	0.589	0.899
Deployment domain images	0.612	0.938
50% COCO and 50% deployment images	0.635	0.935
75% COCO and 25% deployment images	0.634	0.925
90% COCO and 10% deployment images	0.641	0.931

**Table 5:** Object texture results.

Texture Model	${\mathrm{AP}}^{\left[0.5:0.95\right]}$	${\mathrm{AP}}^{0.5}$
Grey base color	0.642	0.931
Random COCO images	0.623	0.946
Random material texture	0.644	0.962
Realistic material texture	**0.653**	**0.963**
Real material texture	0.648	0.948

**Table 6:** Results for additional foreground objects.

Foreground Objects	$n_{\mathrm{FG}}$	Texture	${\mathrm{AP}}^{\left[0.5:0.95\right]}$	${\mathrm{AP}}^{0.5}$
None	0	-	0.653	0.963
YCB tools	U{0,1}	Original	0.657	0.963
YCB tools	U{0,2}	Original	0.653	0.963
YCB tools	U{0,3}	Original	0.660	0.972
YCB tools	U{0,4}	Original	0.659	0.972
YCB tools	U{0,3}	COCO	0.653	0.951
YCB tools	U{0,3}	Random material	0.647	0.958
Cubes	U{0,3}	COCO	0.666	0.987
Cubes	U{0,3}	Random material	**0.669**	**0.989**

**Table 7:** Using real training data.

Model	$n_{\mathrm{TI}}$	${\mathrm{AP}}^{\left[0.5:0.95\right]}$	${\mathrm{AP}}^{0.5}$
Real training images	200	0.709	0.985
PBR training images	5000	0.704	0.989
Pre-trained on PBR and fine-tuned on real images	5000 and 200	**0.785**	**1.00**

**Table 8:** Sequential ablation study on test data with new objects ($AP^{\left[0.5:0.95\right]}$).

Object	Baseline ${}^{1}$	IBL with HDRIs	Realistic Material Texture	Foreground Objects
TB 1	0.620	0.662	0.663	0.677
TB 2	0.481	0.568	0.580	0.629
TB 3	0.466	0.467	0.501	0.556
mean	0.522	0.566	0.581	0.621

^1^ The baseline was trained on 5000 images with tight bounding box computation, white point lights, COCO background images, a single basecolor and no additional 3D objects.

## Data Availability

The turbine blade data are not publicly available due to protection of intellectual property.

## Appendix A

Qualitative results of our object detection model for TB 1 on validation images are shown in Figure A1. Results of our object detection models for TB 1, TB 2 and TB 3 on new test images are shown in Figure A2. Figure A3 shows examples of rendered training images using our image generation methodology.
